# AGREEMENT ON MRI DIAGNOSIS IN COMPRESSIVE MALIGNANT VERTEBRAL FRACTURES

**DOI:** 10.1590/1413-785220233101e258926

**Published:** 2023-04-17

**Authors:** Iranilson Medeiros Germano dos Santos, Carlos Fernando Pereira da Silva Herrero, Raphael de Rezende Pratali, Paulo Moraes Agnollitto, Fernando Figueiredo Waib, Marcello Henrique Nogueira-Barbosa

**Affiliations:** 1 Universidade de São Paulo, Ribeirão Preto Medical School, Department of Medical Imaging, Hematology and Clinical Oncology, Ribeirão Preto, SP, Brazil.; 2 Universidade de São Paulo, Ribeirão Preto Medical School, Department of Orthopedics and Anesthesiology, Ribeirão Preto, São Paulo, Brazil.; 3 Hospital do Servidor Público Estadual, São Paulo, Brazil.

**Keywords:** Spinal Fractures, Magnetic Resonance Imaging, Metastasis, Neoplasm, Osteoporotic Fractures, Spine, Fraturas da coluna vertebral, Imageamento por Ressonância Magnética, Metástase Neoplásica, Fraturas por Osteoporose, Coluna Vertebral

## Abstract

**Objective:**

Verify interobserver and intraobserver agreement of malignant compressive vertebral fractures (MCVF) diagnosis using magnetic resonance imaging (MRI).

**Methods:**

We retrospectively included a lumbar spine MRI of 63 patients with non-traumatic compressive vertebral fracture diagnoses. Each lumbar vertebra was classified as: without fracture, with fracture of benign characteristics, or with fracture of malignant characteristics. Two medical residents in radiology, one musculoskeletal radiologist fellow, one musculoskeletal radiologist, and two spine surgeons evaluated MRI exams, independently and blindly. Each observer performed two readings, with a 15-day interval between evaluations. A simple Kappa coefficient was used to calculate the intra and interobserver agreement. The reference standard classification was based on bone biopsy or clinical, and imaging follow-up of at least two years, for diagnostic performance analysis. Diagnostic performance was assessed by calculating sensitivity, specificity, accuracy, and positive and negative predictive values with a 95% confidence interval (CI).

**Results:**

We observed substantial to perfect intraobserver agreement (kappa: 0.80 to 1.00) and substantial interobserver agreement (kappa 0.64 to 0.77). In general, the sensitivity for the detection of MCVF was moderate, except for the second-year radiology resident that achieved a lower sensitivity. The specificity, accuracy, and negative predictive value were high for all observers.

**Conclusion:**

MCVF diagnosis using MRI showed substantial interobserver agreement. The second-year medical resident achieved lower sensitivity but high specificity for MCVF. Regarding the seniors, there was no statistical significance between spine surgeons and the musculoskeletal radiologist. ***Level of Evidence III; Diagnostic.***

## RESUMO

**Objetivo:**

Verificar a concordância interobservador e intraobservador no diagnóstico de fraturas vertebrais compressivas malignas (MCVF) por meio de ressonância magnética (MRI).

**Métodos:**

Incluiu-se retrospectivamente a ressonância magnética da coluna lombar de 63 pacientes com diagnóstico de fratura vertebral compressiva não traumática. Cada vértebra lombar foi classificada da seguinte forma: sem fratura, com fratura de características benignas ou com fratura de características malignas. Dois médicos residentes em radiologia, um pesquisador radiologista musculoesquelético, um radiologista musculoesquelético e dois cirurgiões da coluna vertebral avaliaram os exames de ressonância magnética, independentemente e cegamente. Cada observador realizou duas leituras, com um intervalo de 15 dias entre as avaliações. O coeficiente Kappa simples foi utilizado para calcular o acordo intra e interobservador. A classificação padrão de referência foi baseada em biópsia óssea ou clínica, e acompanhamento por imagem de pelo menos dois anos, para análise de desempenho diagnóstico. O desempenho diagnóstico foi avaliado através do cálculo de sensibilidade, especificidade, precisão, valores preditivos positivos e negativos com um intervalo de confiança de 95% (IC).

**Resultados:**

Foi observada concordância substancial a perfeita intraobservador (kappa: 0,80 a 1,00) e concordância substancial interobservador (kappa 0,64 a 0,77). Em geral, a sensibilidade para a detecção de MCVF foi moderada, exceto para o segundo ano de residência radiológica que alcançou uma sensibilidade menor. A especificidade, precisão e valor preditivo negativo foram altos para todos os observadores.

**Conclusão:**

O diagnóstico de MCVF por ressonância magnética mostrou uma concordância substancial entre observadores. O residente médico do segundo ano alcançou uma sensibilidade menor, mas alta especificidade para MCVF. Com relação aos veteranos, não houve significância estatística entre os cirurgiões da coluna vertebral e o radiologista músculo-esquelético. ***Nível de Evidencia III; Diagnóstico***.

## INTRODUCTION

The occurrence of non-traumatic fractures in the thoracic and lumbar spine segments is a common problem, especially in elderly individuals, with osteoporosis being the leading cause of these fractures.^1,2^ On the other hand, the spine is also a frequent site of metastatic disease, which can result in pathological fractures.^3^ The etiological diagnosis of these fractures is fundamental since it can modify the therapeutic planning and the prognosis of patients. Failure to diagnose metastatic lesions, or a delay in the diagnosis, may compromise optimal treatment and lead to worse clinical outcomes.^4^

Magnetic resonance imaging (MRI) is considered the gold standard imaging for the differentiation between pathological fractures associated with metastatic lesions and benign osteoporotic fractures.^5-7^ Several MRI signs are described as useful in distinguishing between these fractures, but the interpretation of these signs is subjective, and there are no decisive criteria for diagnosis.^5^ Thus, accurate diagnosis among such fractures based on imaging examinations, even considering experienced radiologists and spinal surgeons may generate doubts.^4^ There is scarce literature on the intraobserver and interobserver agreement in the diagnosis of malignant vertebral compressive fractures (MVCF). To the best of our knowledge, it is not well known whether the medical specialty interferes with the diagnostic performance of MVCF. Thus, the objectives of the present study were to verify intra and interobserver agreement regarding MVCF detection, and to investigate the diagnostic performance of these fractures, comparing radiologists and spine surgeons.

## MATERIALS AND METHODS

### Type of study, population and ethical aspects

This is a retrospective and transverse observational diagnostic study using a database of spine MRI approved by the Institutional Review Board of Ribeirão Preto Medical School, University of São Paulo, Ribeirão Preto, Brazil (Process HCRP n° 13568/2016). Only patients with a previous diagnosis of compressive vertebral fracture secondary to bone insufficiency or malignant disease were included. The cases were searched using the keywords “fracture”, “malignant”, “osteoporotic” and “osteoporosis” in the final impression of lumbar spine MRI radiological reports in the Radiological Information System. Exclusion criteria were a history of chemotherapy, radiotherapy, or surgery before the MRI study and previous history of spinal trauma or infection. The MRI files were anonymized and the confidentiality of the patients’ identity guaranteed in all the study processes.

A total of 220 patients who had the potential to participate in the study were initially enrolled, but after applying the previously mentioned exclusion criteria, 63 patients were included in the study. Lumbar spine MRI of all patients was acquired on the same equipment (1.5 Tesla, Achieva, Philips, Eindhoven, Netherlands). All patients had their diagnosis confirmed either by a histopathological diagnosis or by a clinical and imaging follow-up for at least two years, in cases in which there was no clinical indication of biopsy.

### Image analysis

Two evaluations of the exams in the DICOM format were carried out by two medical residents in radiology second and third year residents (2ndRR and 3rdRR respectively), one musculoskeletal radiologist fellow (MSKRF), one musculoskeletal radiologist with three years of experience in this area (MSKR) and two spine surgeons. Radiology Medical Residents were at the end of their respective training years. The two spine surgeons with seven and eight years of experience were denominated SS7 and SS8, respectively. The observers performed independent and blind evaluations, without knowledge of the final diagnosis of each patient and data on the etiology of the vertebral fracture, as well as without information on the other assessments performed by other physicians.

The evaluation was performed with all the images acquired in the clinical routine, with T2-weighted sagittal, axial and coronal images and T1-weighted sagittal plane images. In some cases, additional sequences were used, such as fat saturation sequences and post-contrast MRI sequences. All observers performed the second evaluation of the images, with a minimum interval of two weeks between the assessments, to investigate intraobserver agreement. In the cases of spine surgeons, before the second evaluation, they were exposed to some scientific articles addressing the issue of diagnostic differentiation between benign osteoporotic and malignant fractures,^7,8^ and it is possible to verify the diagnostic performance before and after the knowledge deepening in the theme. For the analysis of the interobserver agreement, only the first assessment of all observers was used.

The analysis considered only the five lumbar vertebrae of the patients included in the study. In cases that there were lumbosacral transition vertebrae, these were considered as L5 vertebra to make their identification homogeneous. The lumbar vertebral bodies were numbered from caudal to cranial, and each lumbar vertebral body diagnosed as benign osteoporotic fracture, malignant fracture and absence of fracture.

### Statistical analysis

All analysis was performed with SAS software (SAS Institute Inc., Campus Drive Cary, NC, USA) version 9.0. The intra and interobserver agreement were calculated using the simple Kappa coefficient, calculating the confidence intervals (CI) of 95%. We consider the classification proposed by Landis and Koch^9^ in which the Kappa value less than 0.00 is considered poor, between 0 and 0.2 defines slight agreement, between 0.21 and 0.4 fair agreement, between 0.41 and 0.6 moderate agreement, between 0.61 and 0.8 substantial agreement and between 0.81 and 1 almost perfect agreement.

Diagnostic performance was defined calculating sensitivity (SEN), specificity (SP), positive predictive value (PPV), negative predictive value (NPV) and accuracy (ACU) in the diagnosis of malignant fractures, with the respective confidence intervals (CI) of 95%.

## RESULTS

### Sample

**Figure 1:**
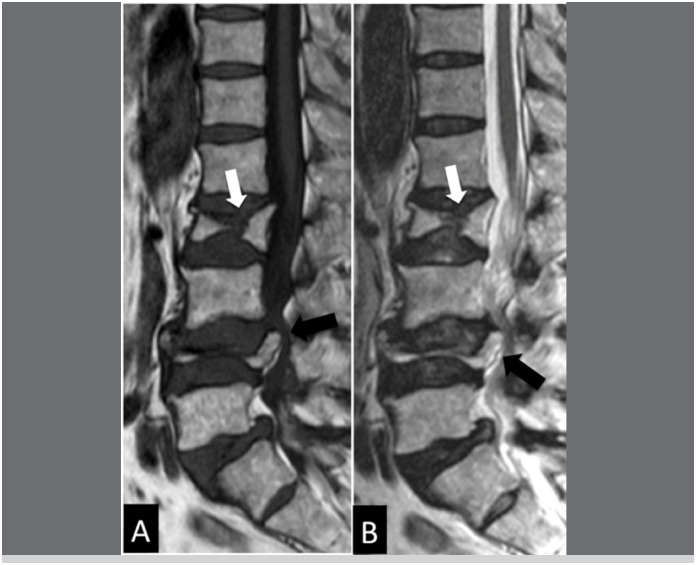
Sagittal T1 (A) and T2 (B) MRI, illustrating a case of bone insufficiency fracture of the L1 and L3 vertebral bodies (presence of 4 lumbar vertebrae and 1 lumbosacral transition vertebra). Notice the retropulsion of the posterior wall bone fragment (black arrows) and the preservation of bone marrow signal (white arrows).

**Figure 2:**
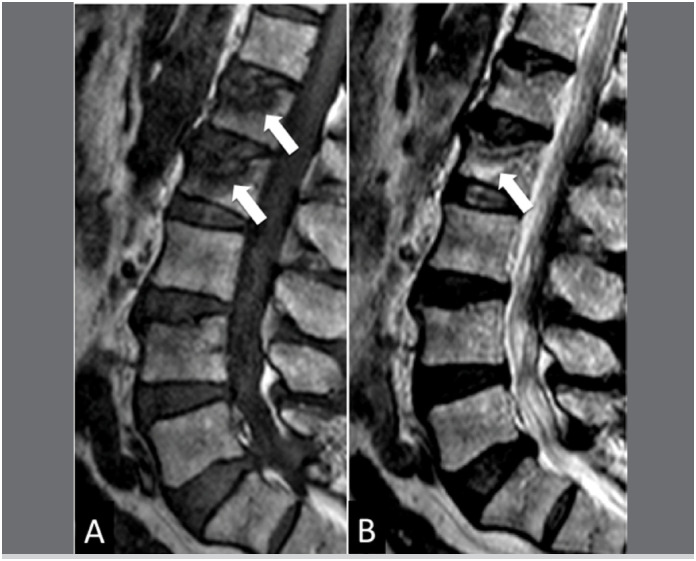
Sagittal T1 (A) and T2 (B) MRIs exemplifying a case of bone insufficiency fracture of the L1 and L2 vertebral body. Observe the low signal band in the fracture trace (white arrows) in addition to preserving the bone marrow signal.

**Figure 3:**
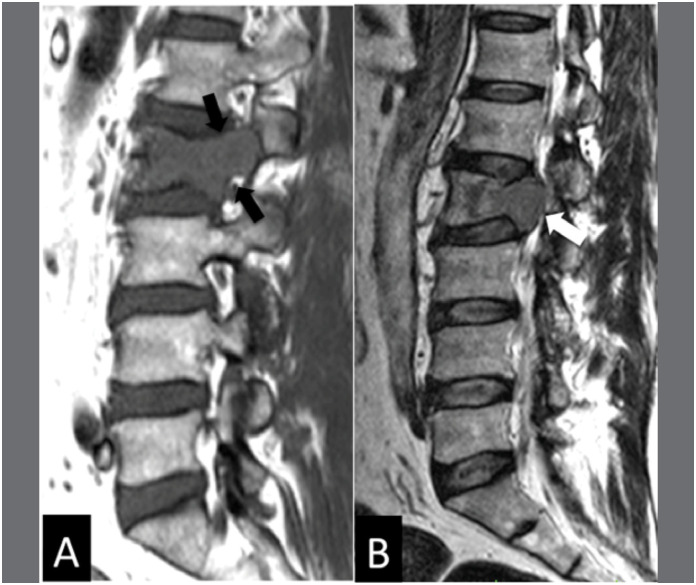
MRI sagittal sequences T1 (A) and T2 (B) exemplifying a case of malignant fracture of the vertebral body of L2. Notice the involvement of the pedicle and the hyposignal throughout the vertebral body (black arrows) and the bulging of the posterior wall (white arrow).

**Figure 4:**
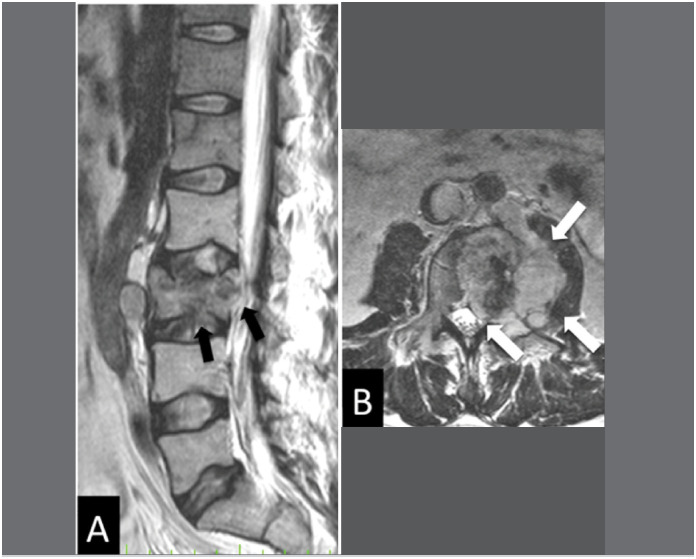
Sagittal T2 (A) and axial T2 (B) sequences illustrating a case of malignant fracture of the vertebral body of L3. Observe the low sign of the bone marrow, the bulging of the posterior wall (black arrows), the formation of epidural and paravertebral mass (white arrows).

Of the 63 studied cases, 38 were women and 25 men with a mean age of 62.2 years. Thirty-three cases had a benign osteoporotic fracture (Figures 1 and 2), and 30 patients presented malignant fractures (Figures 3 and 4). Among the malignant fractures, the majority (12 patients, 40%) was due to multiple myeloma, followed by breast carcinoma (8, 26.7%). The other diagnostics were pulmonary carcinoma (1 patient), prostatic carcinoma (2 patients), oropharyngeal carcinoma (2 patients), cholangiocarcinoma (1 patient) paraganglioma (1 patient), miofibroblastic tumor (1 patient) and leukemia (2 patients).

### Intraobserver agreement

The analysis of intraobserver agreement showed almost perfect agreement between the two evaluations performed by almost all observers (Table 1). Only the surgeon with eight years of experience (SS8) presented substantial intraobserver agreement (Kappa = 0.80) and the second-year radiology resident achieved perfect intraobserver agreement (2ndRR) (Kappa = 1.00). There was no statistically significant difference of intraobserver agreement regarding the comparison between the speciality type and degree, except for the 2ndRR. Among the spine surgeons, an intraobserver agreement was higher for the surgeon with seven years of experience (SS7), but with no statistical significance. Comparing the intraobserver agreement between radiologists and surgeons, it was higher for the senior musculoskeletal radiologist.

**Table 1:** Intraobserver agreement, assessed by the simple Kappa coefficient and its respective confidence intervals (95%).

Observer	Agreement coefficient (Kappa)	Confidence intervals (95%)
2ndRR	1.0	1.0 – 1.0
3rdRR	0.82	0.75 – 0.88
MSKRF	0.91	0.87 – 0.95
MSKR	0.97	0.94 – 0.99
SS7	0.90	0.85 – 0.94
SS8	0.80	0.73 – 0.86

2ndRR – second year radiology resident; 3rdRR – third year radiology resident; MSKRF - radiologist fellow; MSKR – radiologist with 3 years of experience; SS7 – spine surgeon with 7 years of experience; SS8 – spine surgeon with 8 years of experience.

### Interobserver Agreement

Interobserver Agreement analysis used the first evaluation of all observers (Table 2). We did not identify a statistically significant difference between the interobserver agreements of different specialties. The interobserver agreement among all observers, considering the confidence intervals, presented results ranging from moderate to almost perfect. Among the radiologists, the highest interobserver agreement was between the 3rdRR and MSKRF with results ranging from substantial to almost perfect. Among the surgeons, the interobserver agreement ranged from moderate to substantial. Among radiologists and surgeons, in general, the interobserver agreement ranged was substantial on (average from 0.62 to 0.77), and the highest agreement occurred between the 3rdRR and SS8 observers.

**Table 2:** Interobserver agreement, assessed by the simple Kappa coefficient and its respective confidence intervals (95%).

	3rdRR	MSKRF	MSKR	SS7	SS8
2ndRR	0.76 (0.69 – 0.83)	0.64 (0.56 – 0.71)	0.64 (0.57 – 0.72)	0.67 (0.6 - 0.74)	0.74 (0.67 - 0.82)
3rdRR		0.77 (0.71 - 0.84)	0.66 (0.59 - 0.73)	0.72 (0.65 - 0.79)	0.77 (0.7 - 0.84)
MSKRF			0.68 (0.61 - 0.76)	0.64 (0.57 - 0.72)	0.65 (0.58 - 0.73)
MSKR				0.62 (0.55 - 0.69)	0.68 (0.6 - 0.75)
SS7					0.67 (0.59 - 0.74)

2ndRR – second year radiology resident; 3rdRR – third year radiology resident; MSKRF - radiologist fellow; MSKR – radiologist with 3 years of experience; SS7 – spine surgeon with 7 years of experience; SS8 – spine surgeon with 8 years of experience.

### Diagnostic Performance

All values calculated for the evaluation of diagnostic performance are shown in Table 3. Regarding the sensitivity, observers 2ndRR and SS7 presented lower mean values in their first evaluation, but without statistical significance. The SS7 observer showed a significant increase in sensitivity in the second assessment (after the study of the academic articles), still maintaining a mean sensitivity value lower than the other observers, but without statistical significance. The mean values of the sensitivity of the other observers were moderate and similar. The specificity, accuracy and negative predictive value were high for all observers. As shown in Table 3, the PPV ranged from moderate to high values, with no statistically significant difference between the observers.

**Table 3:** Sensitivity (SEN), specificity (ESP), positive predictive value (PPV), negative predictive value (NPV) and accuracy (ACU) analysis, with the respective confidence intervals (CI) of 95%.

	SEN	ESP	PPV	NPV	ACU
2ndRR	36.4 (23.8 - 50.4)	96.5 (93.5 - 98.4)	69.8 (49.2 - 84.7)	87.8 (83.4 - 91.3)	86.1 (82.3 - 89.9)
3rdRR	67.3 (53.3 - 79.3)	96.5 (93.5 - 98.4)	80.4 (66.1 - 90.6)	93.3 (89.6 - 96.0)	91.5 (88.4 - 94.6)
MSKRF	74.5 (61.0 - 85.3)	98.1 (95.6 - 99.4)	89.1 (76.4 - 96.4)	94.8 (91.4 - 97.1)	94.0 (91.4 - 96.6)
MSKR	60.0 (45.9 - 72.9)	96.1 (93.0 - 98.1)	76.7 (61.4 - 88.2)	91.9 (88.0 - 94.9)	89.9 (86.6 - 93.2)
SS7 (E1)	69.1 (55.2 - 80.9)	91.5 (87.5 - 94.6)	63.3 (49.9 - 75.4)	93.3 (89.5 - 96.1)	87.6 (84.0 - 91.2)
SS7 (E2)	70.9 (57.1 - 82.4)	91.5 (87.5 - 94.6)	63.9 (50.6 - 75.8)	93.7 (89.9 - 96.4)	87.9 (84.3 - 91.5)
SS8 (E1)	45.4 (31.9 - 59.4)	94.6 (91.1 - 97.0)	64.1 (47.2 - 78.8)	89.1 (84.8 - 92.5)	86.0 (82.2 - 89.8)
SS8 (E2)	58.2 (44.1 - 71.3)	98.1 (95.6 - 99.4)	86.5 (71.2 - 95.5)	91.7 (87.8 - 94.7)	91.2 (88.1 - 94.3)

2ndRR – second year radiology resident; 3rdRR – third year radiology resident; MSKRF - radiologist fellow; MSKR – radiologist with 3 years of experience; SS7 – spine surgeon with 7 years of experience; SS8 – spine surgeon with 8 years of experience; E1 – first evaluation; E2 – second evaluation

## DISCUSSION

Despite the importance of accurate diagnosis in spinal fractures between benign osteoporotic and malignant, especially in older individuals, several studies suggest that the determination of specific criteria for such a differential diagnosis can be difficult.^5,7,10^ Nevertheless, the influence of the medical specialty on the performance of this differential diagnosis has not been evaluated. Comparing radiologists with spine surgeons, in addition to the experience of these specialists was the objective of the present study. The intra and interobserver agreement rate were also verified and evaluated according to the medical specialty and professional experience time. In the present study, we did not identify a statistically significant difference in diagnostic performance when distinguishing benign osteoporotic from malignant vertebral fractures between the different training levels. The only exception was the low sensitivity obtained for the second year radiology resident that has been less exposed to MRI training. In general, the specificity (always higher than 90%) was considerably higher than sensitivity in the diagnosis of malignant fractures, so, when evaluated by radiologists and spine surgeons, the observation of signs of malignancy are usually consistent with such diagnosis. Kato et al. also observed specificity higher than 85% in 200 fractures evaluated by two spine surgeons.^7^

In the case of spine surgeons, after reading the academic articles on the subject, we noticed that the acquired knowledge was associated with the improvement in diagnostic performance. Therefore, it was observed that the results are better when there is previous knowledge about the characteristic signs in the imaging examinations of benign osteoporotic or malignant vertebral fractures.^7,8^

Regarding the agreement rate, the rates obtained were classified as being substantial to almost perfect for all observers participating in the study. This would suggest a high reproducibility of the evaluation using the diagnostic characteristics commonly attributed to benign osteoporotic and malignant vertebral fractures. Several authors have reported that the interpretation of the characteristic signs for the differential diagnosis is subjective and, thus, the interobserver reproducibility could be quite variable among the studies.^5,7,11-13^

A striking feature of the present study was that the most frequent diagnostic errors were mainly related to cases diagnosed with multiple myeloma. This greater difficulty for diagnosis in cases of fracture associated with multiple myeloma is in agreement with the literature, being that these fractures frequently present characteristics compatible with benignity in MRI.^14^ In the study by Leucovet et al., it was observed that 67% of fractures associated with multiple myeloma had MRI signs characteristic of benign vertebral fractures.^14^ Multiple Myeloma patients comprised 40% of our cases with vertebral fractures secondary to metastasis, and this may explain why average sensitivity achieved for MVCF was just moderate.

Because of the difficulties described here in the differential diagnosis between benign osteoporotic and malignant vertebral fractures, some authors sought to develop instruments that could improve such diagnosis. Recently, a score composed of MRI signs was presented to assist the determination of vertebral fractures by metastases.^7^ The authors reported that with the use of the score described by them, they obtained an accuracy rate of 96.6% in the diagnosis of metastatic malignant fractures. In the present study, in which the observers did not use any specific instrument for diagnosis, the mean accuracy was 90.1%, and the musculoskeletal radiology fellow obtained 94% accuracy. In the article in which the META score was described to assist in the diagnosis of MVCF,^7^ cases with multiple myeloma were excluded, while in the present study they were included.

More recently, Computed Assisted Classification and Machine-learning techniques have been applied to MVCF diagnosis on spine MRI, with promising results.^15-17^ Features derived from Fourier and wavelet transforms, together with the fractal dimension, achieved up to 94.7% of correct classification with the area under the receiver operating characteristic curve (AUC) reaching 0.95.^15^ Neural networks achieved AUC of 0.97 in distinguishing between normal and fractured vertebral bodies, and 0.92 in discriminating between benign and malignant fractures.^16^ A combination of different classification models composing the ensemble to make the final class assignment reached an average value of AUC = 0.94.^17^ Future studies are necessary to confirm artificial intelligence usefulness in the diagnosis of MVCF with external validation.

The present study presents limitations that deserve mention. First, this is a retrospective investigation. Another limitation is that not all cases had histopathological confirmation of the fracture etiology. Cases strongly suggestive of MVCF were biopsied, but in cases that MRI signs favored a benign vertebral fracture, patients were followed clinically and with follow up MRI. All cases had a minimum clinical follow-up of two years from vertebral fracture detection to minimize the risk of including fractures initially identified as osteoporotic fractures but representing a false negative. The classical studies on MVCF also had similar limitation because, in the clinical practice, the biopsy of the vertebral compression fracture is not always necessary or indicated. Therefore, the reference standard for the presence or absence of metastases was based on a best valuable comparator, based on clinical, histologic, biologic, and imaging data.^18-21^

## CONCLUSION

MCVF diagnosis using MRI showed substantial interobserver agreement. The second-year radiology resident achieved lower sensitivity but high specificity for MCVF. Regarding the seniors, there was no statistical significance between spine surgeons and the musculoskeletal radiologist.
